# Genomic epidemiology of the commercially important pathogen *Renibacterium salmoninarum* within the Chilean salmon industry

**DOI:** 10.1099/mgen.0.000201

**Published:** 2018-07-24

**Authors:** Sion C. Bayliss, David W. Verner-Jeffreys, David Ryder, Rudy Suarez, Roxana Ramirez, Jaime Romero, Ben Pascoe, Sam K. Sheppard, Marcos Godoy, Edward J. Feil

**Affiliations:** ^1^​ Milner Centre for Evolution, Department of Biology and Biochemistry, University of Bath, Bath, UK; ^2^​ Weymouth Laboratory, Centre for Environment, Fisheries and Aquaculture Science (Cefas), The Nothe, Weymouth, UK; ^3^​ Laboratorio ETECMA, Puerto Montt, Chile; ^4^​ Facultad de Medicina Veterinaria, Universidad San Sebastian, Puerto Montt 5501842, Chile; ^5^​ Laboratorio de Biotecnología, Instituto de Nutrición y Tecnología de los Alimentos (INTA), Universidad de Chile, Santiago, Chile; ^6^​ Centro de Investigaciones Biológicas Aplicadas (CIBA), Puerto Montt, Chile; ^7^​ Doctorado en Acuicultura, Programa Cooperativo Universidad de Chile, Universidad Católica del Norte, Pontificia Universidad Católica de Valparaíso, Valparaíso, Chile

**Keywords:** aquaculture, epidemiology, whole-genome sequencing, bacterial kidney disease, *Renibacterium salmoninarum*

## Abstract

*Renibacterium salmoninarum* is the causative agent of bacterial kidney disease (BKD), which is a commercially important disease of farmed salmonids. Typing by conventional methods provides limited information on the evolution and spread of this pathogen, as there is a low level of standing variation within the *R. salmoninarum* population. Here, we apply whole-genome sequencing to 42 *R. salmoninarum* isolates from Chile, primarily from salmon farms, in order to understand the epidemiology of BKD in this country. The patterns of genomic variation are consistent with multiple introductions to Chile, followed by rapid dissemination over a 30 year period. The estimated dates of introduction broadly coincide with major events in the development of the Chilean aquaculture industry. We find evidence for significant barriers to transmission of BKD in the Chilean salmon production chain that may also be explained by previously undescribed signals of host tropism in *R. salmoninarum*. Understanding the genomic epidemiology of BKD can inform disease intervention and improve sustainability of the economically important salmon industry. This article contains data hosted by Microreact.

## Data Summary

Short-read data for isolates sequenced as a part of this work have been archived in the EMBL-EBI database under study accession number PRJEB26486 (www.ebi.ac.uk/ena/data/search?query=PRJEB26486). Assemblies have been deposited in the BIGSdb aquaculture database (url - https://pubmlst.org/bigsdb?db=pubmlst_fish_isolates). Metadata and the tree file are available for download from the Microreact project at https://microreact.org/project/SkR7uB10M.

**Impact Statement**

Bacterial kidney disease (BKD) causes a high level of mortality and morbidity in salmonid aquaculture worldwide. The causative agent of BKD, *Renibacterium salmoninarum*, has a slow mutation rate and a highly conserved genome, which limits the discriminatory power of conventional typing techniques such as multi-locus sequence typing and pulsed-field gel electrophoresis. Whole-genome sequencing (WGS) allows for almost all genomic variation to be used for molecular tracing and phylogenetic reconstruction. This technique has been successfully applied to the fine-scale epidemiology of monomorphic human pathogens, such as *Mycobacterium tuberculosis* and *Yersinia pestis*, and a global collection of *R. salmoninarum* [Traynor BJ. *Neuroepidemiology* 2009;33:276–9; Brynildsrud O, Feil EJ, Bohlin J, Castillo-Ramirez S, Colquhoun D *et al. ISME J* 2014;8:746–56]. For the first time, we apply WGS to a collection of *R. salmoninarum* isolates from Chile, a country that hosts the second largest salmon industry in the world and in which BKD imposes a significant commercial burden. We use WGS for fine-scale molecular epidemiology of *R. salmoninarum*, and to place the Chilean isolates within the context of a global isolate collection. Our results point to multiple introductions of BKD into Chile from global sources over a 30 year period, followed by rapid spread within Chile. This study provides a basis for the development of more informed disease management strategies.

## Introduction

Aquaculture is the fastest growing livestock production sector, and has a critical role in the economic welfare of many low- and middle-income countries. Since 2014, the majority of finfish consumed globally are from farmed stocks [1]. However, the rapid expansion and intensification of aquaculture presents serious challenges with respect to sustainability and infectious-disease management. A notorious example is the devastating outbreak of infectious salmon anemia (ISA) virus that swept through Chilean salmon farms in 2007, bankrupting the industry and leaving debts of 1.8 billion US dollars (1.4 billion pounds) [2]. The Chilean Atlantic salmon industry has subsequently recovered, and is now the second largest in the world with an annual production of 502 000 tonnes in 2016 [1]. However, other diseases now pose a major threat to the sustainable development of aquaculture in Chile. One such example is bacterial kidney disease (BKD), caused by the Gram-positive bacillus *Renibacterium salmoninarum*, a chronic granulomatous disease that affects salmonid species worldwide. *R. salmoninarum* was first isolated in Chile from chum salmon (*Oncorhynchus keta*, Walbaum) reared in seawater cages [3], and has gone on to cause disease in Atlantic salmon (*Salmo salar*), rainbow trout (*Oncorhynchus mykiss)* and coho salmon (*Oncorhynchus kisutch*). Treatment of BKD places a significant burden on the industry and currently requires the second largest application of antibiotics in both saltwater and freshwater aquaculture in Chile [4].

Whole-genome sequencing (WGS) provides a powerful approach to tracking the emergence and spread of pathogens [5]. Many of the analytical methods and platforms developed to manage pathogens of relevance to public health can be directly applied to aquaculture [6]. In addition to understanding the epidemiology of *R. salmoninarum*, WGS data can also inform vaccine design and improve understanding of host switching and adaptation. *R. salmoninarum* was the first aquaculture pathogen to which WGS was applied in order to understand evolutionary and epidemiological dynamics [7]. The study revealed a major phylogenetic division within the *R. salmoninarum* population. The data indicated that the pathogen had been introduced to Europe from North America on multiple occasions over the last 50 years via trade in eggs and fry. The data also pointed to frequent host switching of the pathogen between host species.

Despite the commercial burden of *R. salmoninarum* in Chile, the origins, diversity and patterns of spread of this pathogen within this country remain unknown. A recent study used a range of methods to demonstrate that 39 *R. salmoninarum* isolates recovered from four different Chilean salmon farms between 2014 and 2016 were genetically and phenotypically homogeneous [8]. However, to date, the level of genetic heterogeneity within the Chilean population has not been investigated using WGS. Here, we describe an analysis of WGS data for 42 *R. salmoninarum* isolates representing geographically widespread Chilean fish farms. All the isolates were collected as a part of the General Sanitary Mortality Management Program (PSGM) and General Sanitary Management Reproduction Fish Program (PSGR) surveillance programmes. By comparing our data to data from a global collection of *R. salmoninarum* genomes, we identified multiple introductions of the BKD-causing *R. salmoninarum* into Chile. The data also points to a host-restricted lineage, indicating that the frequency of host switching may not always be as high as previously thought. Alternatively, it suggests that other barriers to inter-host transmission may exist in the Chilean fish production chain that have had a strong influence on the spread of *R. salmoninarum* in Chile.

## Methods

### Isolate collection

BKD was listed as a High Risk Disease by the Chilean government as a part of the EAR (Enfermedades de Alto Riesgo) classification in July 9 2013 [9]. A total of 42 *R. salmoninarum* samples were collected and analysed for the current study (Table 1). These isolates were collected as part of two ongoing Chilean surveillance programmes. The General Sanitary Mortality Management Program (PSGM) involves companies and private laboratories performing passive surveillance of diseases according to sanitary/productive criteria [10]. The Servicio Nacional de Pesca (National Fisheries Service) produces sanitary and production PSGM reports at weekly and monthly intervals respectively. The General Sanitary Management Reproduction Fish Program (PSGR) is a programme that mandates screening of all breeding females and has encouraged the voluntary screening of breeding males of all salmonid species since January 24 2003 [11].

**Table 1.** Sample metadata Additional metadata is available for download from the Microreact project at: https://microreact.org/project/SkR7uB10M.

**Sample ID**	**Lineage**	**Host**	**Country**	**Region**	**Year**	**Accession no.**
NCIMB_1111	1	Unknown	USA/UK	Unknown	Not known	ERR327924
Car_96	1	*O. tshawytscha*	USA	Washington	1996	ERR327957
WR99_c2	1	*O. kisutch*	USA	Washington	1999	ERR327960
ATCC_33209	1	*O. tshawytscha*	USA	Oregon	1974	NC_010168.1
D6	1	*O. tshawytscha*	USA	Oregon	1982	ERR327961
NCIMB_2235	1	*O. tshawytscha*	USA	Oregon	1974	ERR327911
GR5	1	*T. thymallus*	USA	Montana	1997	ERR327959
05372K	1	*O. tshawytscha*	USA	Grande Ronde Basin, Oregon	2005	ERR327906
Cow-chs-94	1	*O. tshawytscha*	USA	Cowlitz River, Washington	1994	ERR327915
Carson_5b	1	*O. tshawytscha*	USA	Confluence Tyee Creek and Wind River, Washington	1994	ERR327905
99326	1	*O. mykiss*	UK	Wales, site Y	1999	ERR327938
99332	1	*O. mykiss*	UK	Wales, site Y	1999	ERR327943
99329	1	*O. mykiss*	UK	Wales, site X	1998	ERR327937
99333	1	*O. mykiss*	UK	Wales, site X	1998	ERR327921
99345	1	*O. mykiss*	UK	Wales, site X	1998	ERR327948
MT444	1	*S. salar*	UK	Western Isles, Scotland	1988	ERR327916
MT1470	1	*O. mykiss*	UK	Tayside, Scotland	1994	ERR327910
MT1511	1	*O. mykiss*	UK	Strathclyde, Scotland, site B	1994	ERR327914
MT3315	1	*O. mykiss*	UK	Strathclyde, Scotland, site B	2008	ERR327928
MT1363	1	*O. mykiss*	UK	Strathclyde, Scotland	1993	ERR327920
MT1880	1	*S. salar*	UK	Strathclyde, Scotland	1996	ERR327909
MT3106	1	*O. mykiss*	UK	Strathclyde, Scotland	2006	ERR327939
MT3482	1	*S. salar*	UK	Strathclyde, Scotland	2009	ERR327934
MT3483	1	*S. salar*	UK	Strathclyde, Scotland	2009	ERR327941
MT3479	1	*S. salar*	UK	Orkney, Scotland	2008	ERR327933
MT1262	1	*S. salar*	UK	Highlands, Scotland	1992	ERR327922
MT1351	1	*S. salar*	UK	Highlands, Scotland	1993	ERR327904
MT2943	1	*S. salar*	UK	Highlands, Scotland	2005	ERR327936
MT2979	1	*O. mykiss*	UK	Highlands, Scotland	2005	ERR327935
MT839	1	*S. salar*	UK	Highlands, Scotland	1990	ERR327917
MT452	1	*O. mykiss*	UK	Dumfries and Galloway, Scotland, site A	1988	ERR327918
MT3277	1	*O. mykiss*	UK	Dumfries and Galloway, Scotland, site A	2008	ERR327926
MT3313	1	*O. mykiss*	UK	Central Scotland	2008	ERR327925
5007	1	*O. mykiss*	UK	Scotland	2005	ERR327923
MT239	1	*S. salar*	UK	Scotland	1988	ERR327913
MT861	1	*S. salar*	UK	Scotland	1990	ERR327919
9025	1	*O. mykiss*	UK	Yorkshire, England	2009	ERR327912
96071	1	*O. mykiss*	UK	Hampshire, England, site Z	1996	ERR327927
99341	1	*O. mykiss*	UK	Hampshire, England, site Z	1998	ERR327949
99344	1	*O. mykiss*	UK	Hampshire, England	1998	ERR327940
1205	1	*O. mykiss*	UK	–	2001	ERR327930
7105	1	*O. mykiss*	UK	–	2007	ERR327932
99327	1	*O. mykiss*	UK	–	1997	ERR327931
7439	1	*S. salar*	Norway	Sognefjorden, Sogn og Fjordane	1984	ERR327971
6863	1	*O. mykiss*	Norway	Osterøy, Hordaland	2009	ERR327965
5223	1	*S. salar*	Norway	Kvinnherad, Hordaland	2005	ERR327964
6553	1	*S. salar*	Norway	Hemne, Sør-Trøndelag	2008	ERR327955
6642	1	*S. salar*	Norway	Hemne, Sør-Trøndelag	2008	ERR327956
6694	1	*O. mykiss*	Norway	Hemne, Sør-Trøndelag	2008	ERR327962
6695	1	*O. mykiss*	Norway	Hemne, Sør-Trøndelag	2008	ERR327968
Rs_8	1	*S. salar*	Canada	New Brunswick	2008	ERR327944
Rs_10	1	*S. salar*	Canada	New Brunswick	2009	ERR327945
Rs_2	1	*S. salar*	Canada	New Brunswick	2005	ERR327951
Rs_3	1	*S. salar*	Canada	New Brunswick	2005	ERR327947
Rs_4	1	*S. salar*	Canada	New Brunswick	2006	ERR327946
Rs_5	1	*S. salar*	Canada	New Brunswick	2007	ERR327950
Rs_6	1	*S. salar*	Canada	New Brunswick	2007	ERR327953
BPS_91	1	*O. gorbuscha*	Canada	Nanaimo, British Columbia	1991	ERR327952
BQ96_91–1	1	*O. kisutch*	Canada	Nanaimo, British Columbia	1996	ERR327963
5006	1	*O. kisutch*	Canada	Bella Bella, British Columbia	1996	ERR327942
DR143	1	*S. fontinalis*	Canada	Alberta	1972	ERR327954
NCIMB_1114	2	*S. salar*	UK	River Dee, Scotland	1962	ERR327908
NCIMB_1116	2	*S. salar*	UK	River Dee, Scotland	1962	ERR327907
7448	2	*S. salar*	Norway	Stranda, Møre og Romsdal	1986	ERR327970
7441	2	*S. salar*	Norway	Storfjord, Møre og Romsdal	1985	ERR327966
7449	2	*S. salar*	Norway	Skjervøy, Troms	1987	ERR327969
684	2	*S. trutta*	Norway	Aurland, Sognefjorden, Sogn og Fjordane	1987	ERR327958
7450	2	*S. salar*	Norway	Askøy, Hordaland	1987	ERR327967
Ch2	1a	*S. salar*	Chile	XI	2014	ERS2444820
Ch41	1c	*O. mykiss*	Chile	XII	2016	ERS2444846
Ch3	1b	*S. salar*	Chile	X	2013	ERS2444838
Ch27	1c	*S. salar*	Chile	XII	2016	ERS2444843
Ch30	1a	*S. salar*	Chile	XII	2015	ERS2444817
Ch1	1a	*S. salar*	Chile	XI	2015	ERS2444819
Ch9	1c	*O. kisutch*	Chile	XI	2013	ERS2444851
Ch22	1a	*S. salar*	Chile	X	2013	ERS2444827
Ch33	1a	*S. salar*	Chile	X	2012	ERS2444825
Ch16	1b	*S. salar*	Chile	X	2012	ERS2444837
Ch21	1d	*S. salar*	Chile	VIII	2014	ERS2444844
Ch36	1a	*S. salar*	Chile	XI	2016	ERS2444823
Ch6	1a	*O. kisutch*	Chile	X	2013	ERS2444847
Ch10	1b	*S. salar*	Chile	X	2012	ERS2444840
Ch18	1b	*S. salar*	Chile	X	2013	ERS2444839
Ch25	1b	*O. kisutch*	Chile	X	2013	ERS2444850
Ch24	1a	*S. salar*	Chile	XII	2015	ERS2444818
Ch37	1b	*O. mykiss*	Chile	X	2013	ERS2444845
Ch34	1a	*S. salar*	Chile	XI	2016	ERS2444824
Ch20	1a	*S. salar*	Chile	XI	2016	ERS2444822
Ch40	1b	*S. salar*	Chile	XI	2016	ERS2444836
Ch29	1a	*O. kisutch*	Chile	X	2013	ERS2444848
Ch13	1a	*O. kisutch*	Chile	X	2013	ERS2444849
Ch31	1c	*O. kisutch*	Chile	X	2015	ERS2444856
Ch23	1c	*O. kisutch*	Chile	X	2015	ERS2444857
Ch26	1c	*O. kisutch*	Chile	X	2015	ERS2444858
Ch35	1c	*O. kisutch*	Chile	X	2013	ERS2444854
Ch39	1c	*O. kisutch*	Chile	X	2015	ERS2444853
Ch19	1a	*S. salar*	Chile	IX	2013	ERS2444831
Ch28	1a	*S. salar*	Chile	IX	2012	ERS2444835
Ch7	1a	*S. salar*	Chile	IX	2015	ERS2444829
Ch15	1a	*S. salar*	Chile	IX	2015	ERS2444830
Ch14	1a	*S. salar*	Chile	IX	2013	ERS2444834
Ch5	1a	*S. salar*	Chile	IX	2013	ERS2444832
Ch8	1a	*S. salar*	Chile	IX	2013	ERS2444833
Ch11	1a	*S. salar*	Chile	X	2012	ERS2444828
Ch38	1a	*S. salar*	Chile	X	2016	ERS2444826
Ch4	1c	*O. kisutch*	Chile	X	2015	ERS2444852
Ch42	1c	*O. kisutch*	Chile	X	2016	ERS2444855
Ch32	1a	*S. salar*	Chile	XI	2015	ERS2444821
Ch17	1b	*S. salar*	Chile	IX	2013	ERS2444841
Ch12	1b	*S. salar*	Chile	IX	2013	ERS2444842

### WGS

*R. salmoninarum* isolates were grown on KDM-2 solid medium [12] at 15 °C, with single colonies being selected and grown for DNA extraction. Paired-end sequencing was performed on the Illumina MiSeq platform with a read length of 300 bp. Short reads have been deposited in the ENA archive under project accession number PRJEB26486 (Table 1). Short-read sequences from Brynildsrud *et al.* [7] were included as a reference collection and analysed using the same tools and settings detailed below [7].

### Mapping and variant calling

Reads from the current study alongside those from the reference collection were mapped to the reference genome ATCC 33209 using smalt 0.7.6 (default settings) and a mean insert size of 300 bp [13]. Reads containing insertions or deletions were realigned using the Genome Analysis Toolkit's Indel Realigner 3.2.2 [14]. Single nucleotide polymorphisms (SNPs) were called using SAMtools 0.1.18 [15]. Variants were filtered using in-house scripts to include only SNPs with >4× read depth per base (>2 per strand), >95 % read support for the alternative variant and mapping quality >30. All bioinformatics analyses were performed using MRC CLIMB [16].

### Phylogenetic analysis

The consensus sequences from aligning short reads to the complete genome sequence of ATCC 33209 were used to reconstruct the phylogeny of *R. salmoninarum.* Regions in repetitive tracts of DNA, or within 50 bp of a repetitive region as identified by Nucmer, were masked from downstream analyses [17]. A maximum-likelihood (ML) tree using RAxML v8.2.10 produced trees that exhibited poor bootstrap support values at multiple nodes [18]. Initial observation of a distance matrix of the SNP-based alignment indicated that a number of isolates shared zero SNP differences and visualization of the bootstrap tree using treespace indicated that isolates with zero SNP distances were being variably positioned within the phylogeny tree, negatively impacting on the reproducibility of the tree [19]. All isolates with zero SNP distances were clustered together and a representative sequence was selected from the cluster using the isolate with the lowest number of missing sites from an alignment of variable sites [20]. The date of the earliest isolate from each cluster was used for subsequent dating analyses. Removal of the redundant sequences produced an alignment of 85 single genome and representative sequences for downstream analysis. There were no recombination regions identified in the data set by either ClonalFrameML 1.11 or Gubbins 2.3.1 [21, 22]. ML trees were reconstructed using a rapid bootstrap analysis best-scoring tree search in RAxML with 100 bootstraps to generate support values [18]. Trees and figures were visualized using ggtree 1.09.00 and ggplot 2.2.1 in the R software environment [23, 24].

### Establishing the timescale of disease emergence

The root-to-tip distance of the ML tree produced by RAxML was analysed using TempEst 1.5.1 (supplementary analysis) using the heuristic residual mean squared value to assess regression fit [25]. Root-to-tip analysis indicated that there was a clock-like signal within the data set. Therefore, beast was used to further investigate the temporal signal within the data [26].

The data set containing both single isolate genomes and genome sequences representative of multiple isolates detailed previously was used for tip-based divergence dating. Genome sequences that represented multiple identical genome sequences (0 pairwise SNP differences) sampled at different times were assigned the date of isolation of the oldest isolate. An initial beast run was performed to test for deviations from a strict clock using an uncorrelated log normal relaxed clock model and a Bayesian Skyline tree prior (exponential prior, mean=10, stdev=0.3337). Three individual runs were performed and these converged to produce comparable parameter estimates. The standard deviation from the mean of the uncorrelated lognormal relaxed clock was small (mean of combined runs: 0.449), indicating that there was low variation in rates among branches. When this parameter takes values greater than 1 then the standard deviation in branch rates is greater than the mean rate, and the data is considered to exhibit substantial rate heterogeneity among lineages. This was considered sufficient evidence to test the assumptions of a strict molecular clock (normal prior, mean=3.324×10^–4^, sigma=0.477×10^–4^) on the data using a prior estimate of 3.324×10^–4^ mutations per site per year as calculated by previous authors [7]. Both strict and relaxed clock models were performed using the general time reversible (GTR) model of nucleotide substitutions (exponential prior with an alpha of 2 and beta of 0.5) with a Bayesian skyline tree model (Jeffreys prior). The Markov chain Monte Carlo (MCMC) value was sampled every 1000 of 200 000 000 iterations. The tree produced by these analyses with the highest maximum clade credibility was annotated with the dates and associated confidence intervals (CIs) assigned to each node. Each model was repeated in three independent runs and compared to ensure consistently good mixing and consensus results. In addition to this, a run sampling only from the prior was performed for both analyses to ensure that none of the estimates were expressions of the priors. The relaxed clock was applied to downstream analysis as previous authors suggest that it represents real phylogenies more accurately than a strict clock model [27].

## Results and Discussion

### Phylogenetic analysis reveals multiple lineages of *R. salmoninarum* in Chile

**Fig. 1.**
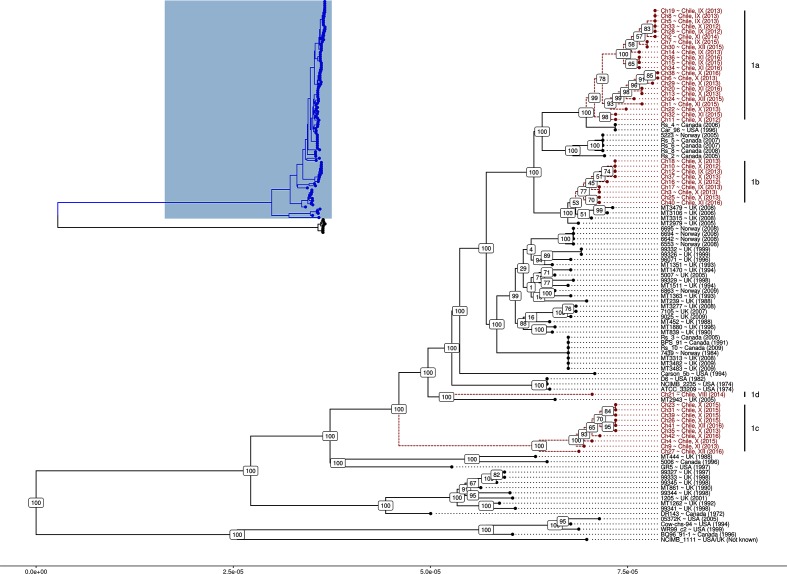
ML phylogenetic tree of 110 *R. salmoninarum* genomes. Short reads were mapped against reference genome ATCC 33209 and consensus sequence alignments were passed to RAxML for phylogenetic reconstruction. Inset: phylogenetic tree of all *R. salmoninarum* isolates. Clade 1 isolates are highlighted in blue. Main panel: phylogenetic tree of *R. salmoninarum* clade 1. The multi-isolate Chilean lineages are labelled. Chilean isolates are highlighted in red. Bootstrap values are shown at each node.

The phylogenetic analysis of WGS sequences from 42 Chilean *R. salmoninarum* isolates, combined with the global collection from Brynildsrud *et al.* [7] is presented in Fig. 1. A Microreact project has been created for the data presented in Fig. 1, which allows for visualization of the tree and associated metadata in an interactive user interface [28]. The Microreact project is available at https://microreact.org/project/SkR7uB10M. Whole-genome assemblies have been deposited in the BIGSdb aquaculture database (https://pubmlst.org/fish/).

The phylogenetic tree confirms two deep-branching clades within the *R. salmoninarum* population, as noted by Brynilsrud *et al*., with little intra-clade heterogeneity [7] (Fig. 1, inset). All of the Chilean isolates sequenced as a part of the present study were found within the much more deeply sampled, aquaculture-associated clade 1 [7]. This clade contains nearly all of the known genome sequences of *R. salmoninarum* recovered from salmonids from North America and Europe. Clade 1 has low genetic heterogeneity, with less than 500 SNP differences between the most distant isolates within the clade. This observation broadly supports a recent study that used a range of methods to demonstrate that 39 *R. salmoninarum* isolates recovered from four different Chilean salmon farms between 2014 and 2015 were genetically and phenotypically homogeneous [8].

**Fig. 2.**
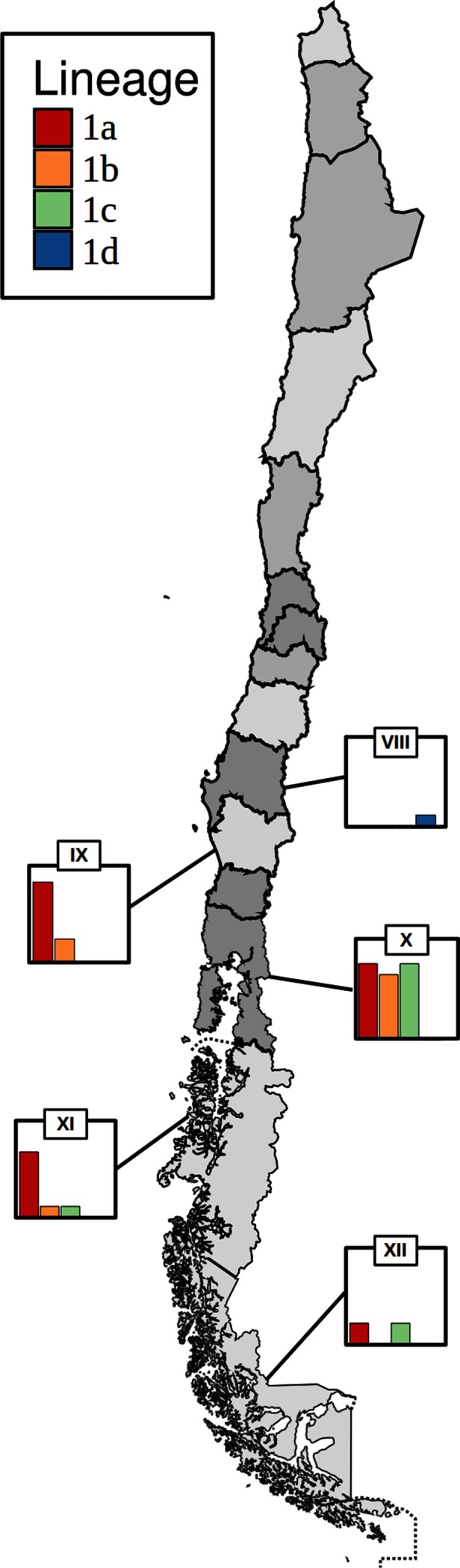
A map of Chile with administrative regions highlighted. The five regions from which *R. salmoninarum* isolates were collected as part of the current study are indicated. Bar charts show the number of isolates collected from each lineage in each region. The maximum value of the *y*-axis in all bar charts is seven.

The Chilean isolates belong to three multi-isolate lineages (1a, 1b, 1c) and one singleton lineage (1d) (Fig. 1; Microreact – https://microreact.org/project/SkR7uB10M). The nomenclature 1a, 1b, 1c and 1d is used here to describe the Chilean *R. salmoninarum* lineages, but these groupings are distinct from those used by previous authors to delineate phylogenetic groupings within clade 1 [7]. None of these clusters contained isolates from outside of Chile, providing initial evidence that there has been very limited transmission of the pathogen from Chile to other countries. However, the data reveal that each of the lineages, except for the singleton lineage 1d, have spread between multiple administrative regions within Chile (Fig. 2). Lineage 1a is the largest of the three Chilean lineages and contains 22 isolates sampled between 2012 and 2016 from the three central regions (IX, X and XI) and one southern region (XII). Lineage 1b contains nine isolates collected from three regions (IX, X and XI) in central Chile between 2012 and 2016. Lineage 1c contains ten isolates collected from central and southern regions X, XI and XII between 2013 and 2016. The single representative isolate from lineage 1d was recovered from region VIII in 2014.

Within each lineage, the Chilean isolates are more closely related to one another than to isolates from other countries, consistent with clonal dissemination within Chile. However, two of the Chilean lineages share a recent common ancestor with isolates from other countries; thus, indicating potential sources of these introductions. Chilean lineage 1a is closely related, and positioned immediately proximal to, two North American isolates, Car96 and Rs_4. A cluster of four Canadian isolates (plus a single exceptional Norwegian isolate) are positioned immediately basal to those two North American isolates. This points to a North American origin of lineage 1a. Similarly, lineage 1b is immediately proximal to four isolates from Scotland, consistent with a transmission from Scotland to Chile. There were no isolates within the collection immediately basal to lineage 1c. Strains GR5 (USA), MT444 (UK) and 5006 (Canada) were located basally, but were too distant to suggest a recent introduction from these sources. The singleton representative isolate of lineage 1d does not cluster with any of the other isolates, Chilean or otherwise, within the collection. Both lineage 1c and 1d are sufficiently distant from other isolates within the collection that no inference of transmission source can be drawn. These lineages likely represent introductions into Chile from source populations that have not been sampled within the current study, such as those in Asia.

### Spread of introduced lineages within the Chilean aquaculture network

**Fig. 3.**
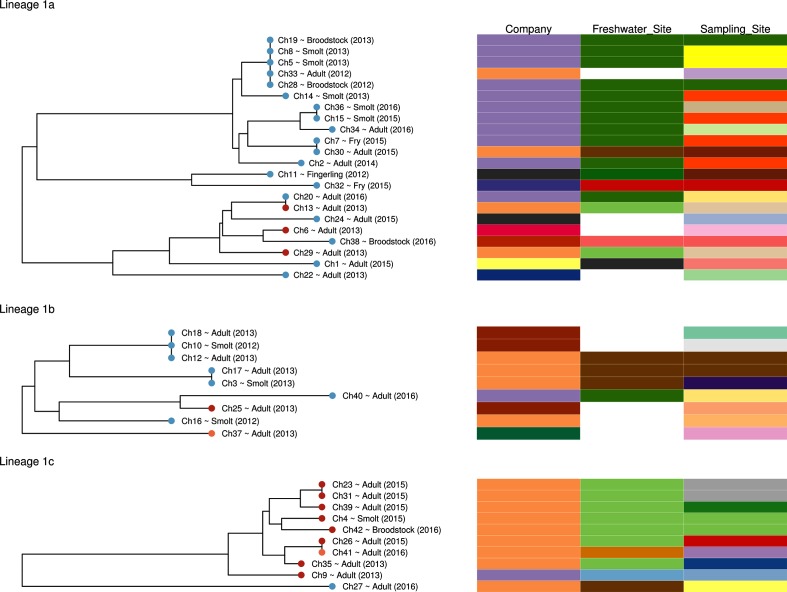
Sampling site and history within the food production network for all Chilean isolates. A timed phylogeny for each lineage is presented on the left. Tip labels contain the life stage of the host animal and the year in which a sample was taken. Tips are coloured by host species (Atlantic salmon, blue; rainbow trout, orange; coho salmon, red). The company, hatchery and point of sample origin are shown as coloured columns to the right of the phylogeny. Each unique company, hatchery and farm site is designated with a unique colour. In those cases in which the colour provided for the point of origin matches the colour provided for the hatchery then this indicates that the sample was taken in the hatchery. Metadata fields for which there is no data were left blank.

Raising of salmonids for farming is typically separated into at least two stages. Eggs are produced from managed breeding lines (broodstock), hatched and raised to a juvenile stage (smolt) in freshwater hatcheries, before being transferred to seawater sites to ‘grow-out’ until the adult animal is of sufficient size for harvesting. Information on the stage in the food production chain at which a diseased individual was sampled was available for the majority of the Chilean isolates as given in the Microreact project (Fig. 3). This included anonymized information on the company managing the hatcheries and farm sites, the freshwater hatchery at which it was raised and the point of sampling, whether that was a freshwater hatchery for broodstock, fingerlings and smolt or a seawater/freshwater farm site for post-smolt to adult fish.

There is no clear clustering of strains or lineages by company or freshwater or saltwater site (Fig. 3; Microreact – https://microreact.org/project/SkR7uB10M). Each of the lineages was sampled from hatchery or farm sites of two or more companies. In three cases, a cluster of effectively identical isolates, such as Ch7 and Ch30, were sampled from sites owned by two separate companies. Moreover, phylogenetically distinct lineages were observed to have been sampled concurrently from the same site, for example multiple isolates from lineage 1a and a single isolate from lineage 1b (Ch40) were collected from freshwater site 1 (Fig. 3, dark green) in 2016. This indicates that there has been frequent transmission between sites owned by different companies, and our analysis has not been overly biased by over-sampling from specific sites or stages of the food production process.

It is unknown at which stage in the food production chain *R. salmoninarum* infection is acquired. Highly similar strains were observed in isolates raised on the same hatchery sites over consecutive years, such as isolates Ch8, Ch5, Ch19 and Ch28 between 2012–2013 (Fig. 3, light green), suggesting related dominant strains, private to individual sites, persist from year to year. Conversely, *R. salmoninarum* from diseased fish sampled from hatchery site 6 and from farm sites stocked by smolt from site 6 were found to be representatives of all three major Chilean lineages (1a, 1b and 1c). These data suggest that hatchery sites may be colonized by multiple unrelated lineages. An alternative explanation would be that strains contributing to outbreaks on farms represent a mixture of strains acquired from both farm and hatchery sites. Further sampling of ‘house strains’ within individual hatchery and farm sites would be required to quantify to what extent isolates acquired in seawater or freshwater farm sites contribute to disease in the field.

### Limited host association amongst Chilean lineages

*R. salmoninarum* has been previously shown to have a high rate of host switching, with genetically identical, or near-identical, isolates capable of causing disease in multiple host species [7]. The data presented here broadly support this, as each of the Chilean lineages co-occurred in multiple host species. Lineage 1a was predominantly from Atlantic salmon (19/22), with the remaining three strains isolated from coho salmon. One of the coho salmon isolates, Ch13, was genetically identical to a strain isolated from Atlantic salmon, Ch20, suggesting a very recent host transmission. The three coho salmon isolates also clustered closely with five Atlantic salmon isolates. Lineage 1b was also predominantly from Atlantic salmon hosts (7/9), with single strains from rainbow trout and coho salmon. In contrast, lineage 1c was predominantly (8/10) isolated from coho salmon, with single isolates from Atlantic salmon and rainbow trout. Lineage 1c is significantly associated with a coho salmon host when compared the rest of the collection (Table S1, available in the online version of this article; Fishers exact *P*=<0.005). Despite this, lineage 1c contains another example of a very recent inter-host transmission, as rainbow trout isolate Ch41 is genetically identical to coho salmon isolate Ch26.

The association between lineage 1c and coho salmon may imply a previously undescribed signal of host association in *R. salmoninarum.* However, it seems likely that the clustering of isolates from coho salmon in lineage 1c is a product of the surveillance programme sampling efforts, as all but one of the coho salmon isolates in lineage 1c were collected from farms supplied by a single hatchery or from the hatchery itself (FW3, Fig. 3; Microreact - https://microreact.org/project/SkR7uB10M). The only example of lineage 1c in Atlantic salmon was also from a site owned by the same company. Therefore, lineage 1c may have had limited opportunity to spread outside of the immediate production network since its initial introduction. However, farm sites in Chile often contain multiple farmed species in close proximity, which would provide many opportunities for host-switching. The presence of isolates Ch41 and Ch27, from rainbow trout and Atlantic salmon, respectively, suggests that lineage 1c has the capacity for inter-host transmission.

### Dating the introductions of *R. salmoninarum* into Chile

**Fig. 4.**
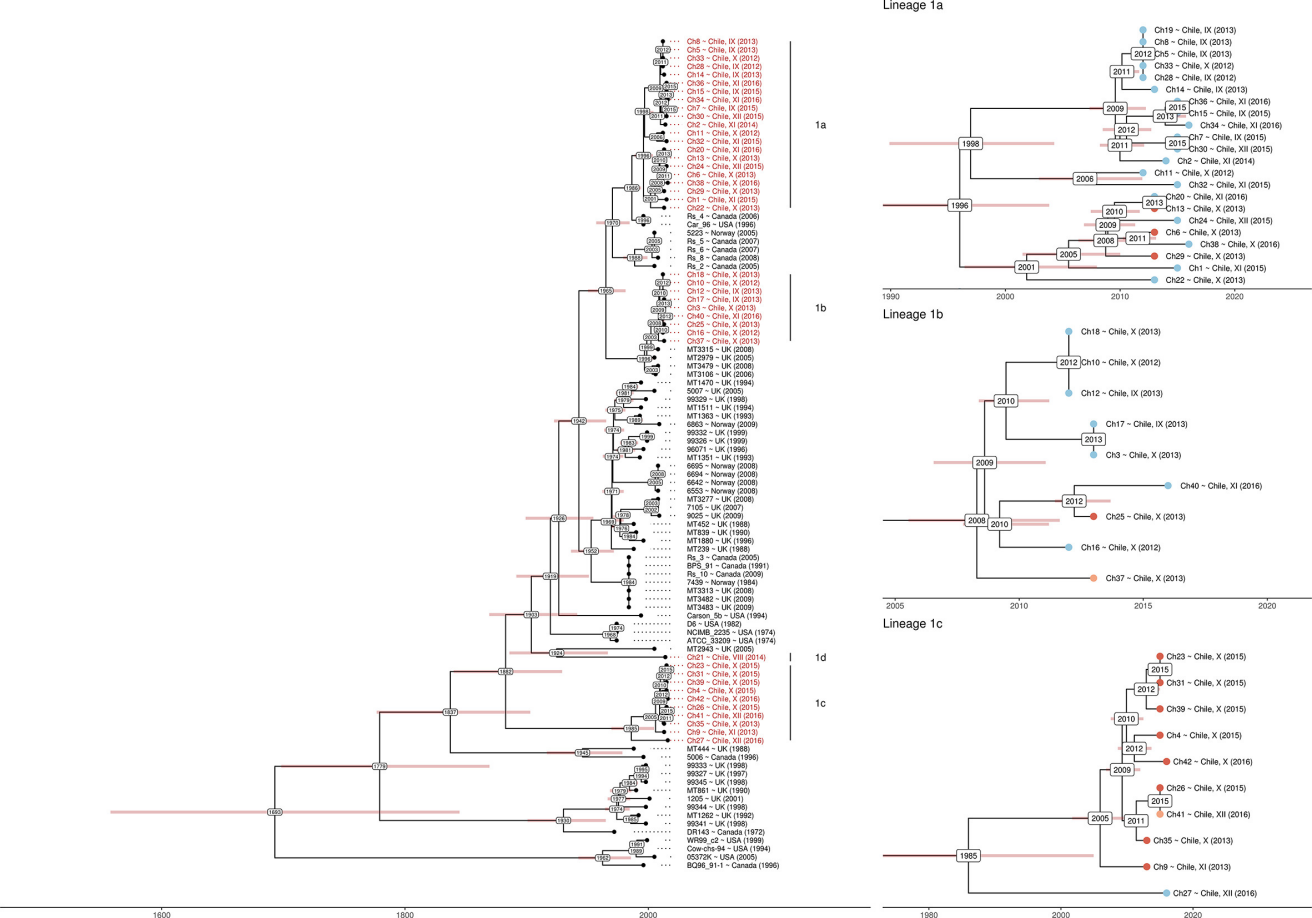
beast reconstruction of the tip-dated phylogenetic tree of *R. salmoninarum* clade 1 using a relaxed-clock model. Short reads were mapped against reference genome ATCC 33209 and consensus sequence alignments were passed to beast for phylogenetic reconstruction. Left panel: phylogenetic tree of all *R. salmoninarum* clade 1 isolates. Chilean isolates are highlighted in red. Chilean multi-isolate lineages are labelled. Tip labels of Chilean isolates are highlighted in red. Right panel: phylogenetic trees of the three Chilean multi-isolate lineages (1a, 1b, 1c). Tips are coloured by host species (Atlantic salmon, blue; rainbow trout, orange; coho salmon, red). Estimated divergence dates are annotated at each node and 95 % CIs indicated (red bar).

Having described four introductions of *R. salmoninarum* to Chile, three of which resulted in significant onward spread within Chile, we used the WGS data to estimate the dates of these international transmission events. The low rate of recombination in this species facilitates such an analysis, and leads to more clock-like accumulation of mutations [7]. A root-to-tip analysis of a ML phylogeny confirmed a significant temporal signal within the *R. salmoninarum* data set (supplementary analysis, Fig. S1). In order to estimate the rate of molecular evolution, beast2, a software platform for Bayesian analysis of molecular sequences using the Markov chain Monte Carlo (MCMC) method, was applied to a tip-dated phylogeny to generate a tree with estimates of divergence dates assigned to the nodes within the phylogeny [26]. Application of a relaxed clock model suggested that there was a clock-like signal within the data (standard deviation from the mean=0.449). The results of a relaxed and strict clock analysis produced similar estimates of divergence dates (Table S2, Fig. 4). The mutation rate predicted by the relaxed clock model was 2.09×10^−7^ mutations per genome per year, which is only marginally slower than the previous estimate from the global data alone of 3.79×10^−7^ mutations per genome per year [7] (Table S2). These values are also comparable to mutation rate estimates from monomorphic, human pathogens such as *Mycobacterium tuberculosis* [29]. The slight decrease in our estimate from the previous estimate for this species may be attributed to the removal of SNPs from repetitive regions in the current study. This produced 3504 variable sites compared to 3600 in the previous study. Repetitive regions are both more likely to contain false positive SNPs due to problematic mapping in repetitive sequences and also to contain horizontally acquired mobile genetic elements that have the potential for higher mutation and recombination rates [30]. It was observed during the root-to-tip regression analysis that older isolates had, on average, more mutations than the model expectation. This may be due to mutations accrued during the long-term storage and sub-culturing of historical isolates. We did not account for this bias in the current work, but incorporation of this effect into the clock model should be a consideration for future researchers working with historical isolates.

The date for the introduction of lineage 1a was estimated as 1996 (95 % CI: 1988–2003), lineage 1b as 2008 (95 % CI: 2005–2011) and lineage 1c as 1986 (95 % CI: 1967–2002) (Fig. 4, Table S2). The estimated date of lineage 1c coincides with the rise of large-scale commercial aquaculture in Chile in the 1980s (Fig. S2). The first confirmed report of outbreaks of BKD in Chile was in 1983 in saltwater net pen reared *Oncorhynchus* from region XI [3]. Further outbreaks were subsequently confirmed in 1984 in the southern region X hatcheries. The current analysis would suggest that lineage 1c isolates in the current study represent descendants of this original outbreak that have diversified and spread around the network of farms that raise coho salmon, with limited spread outside of this network. The first salmonids introduced to Chile were *Oncorhynchus* species from the USA and Japan that were introduced as part of ranching programmes [31]. These were hatchery raised and released into rivers. As the current collection contains no isolates from Japan and few isolates from the USA, we are unable to support or refute a North American or Japanese origin of the initial 1980s outbreaks of BKD in Chile.

The tree dating analysis suggests that lineages 1a (1996) and 1b (2008) were introduced after the rise of Atlantic salmon aquaculture, and that ongoing introductions of *R. salmoninarum* from other countries further contributed to cases of BKD in Chile. The estimated introduction date of lineage 1a in ~1996 (likely from North America) coincides with the shift in the Chilean aquaculture industry from coho salmon to Atlantic salmon production in the mid-1990s (Fig. S2) [32]. Consistent with this, lineage 1a was predominantly identified in Atlantic salmon stock. The previous analysis suggests that lineage 1b was introduced at a more recent date coinciding with the major outbreaks of ISA in Chile in 2007 [33]. The phylogenetic analysis suggests that lineage 1b is closely related to isolates recovered from Atlantic salmon farmed in Scotland. Although there is some dispute, studies have implicated transfer of Atlantic salmon ova from Europe as a likely source of introduction of the virus ISA into Chile [34]. Indeed, the volume of imported Atlantic salmon ova peaked in 2008, with the two largest exporters being Norway (149 710 000 eggs) and Scotland (55 889 500 eggs) (Fig. S3). These data are, thus, consistent with the evidence provided by the WGS data in support for the introduction of lineage 1b into Chile from Scotland in around 2008.

The source and date of introduction of lineage 1d into Chile is unclear. The tree dating analysis estimates the date at which lineage 1d (5853) diverged from the most recent common ancestor of the most closely related isolate in the collection (MT2943) as 1924 (95 % CI: 1883–1964), which could represent a pre-industrial introduction of *R. salmoninarum* to Chile. Alternatively, the isolate could have arisen from a geographically-distinct source population that was not sampled as a part of the current collection. There is currently insufficient evidence to test either hypothesis, but we consider the latter hypothesis to be more likely.

### Conclusion

Our WGS analysis reveals that there have been at least four introductions of *R. salmoninarum* into Chile, three of which resulted in substantial onward spread. All four of these Chilean lineages arose from within clade 1, which has previously been described as a successful, aquaculture-associated, clade that has spread internationally. As there are no salmon species native to South America, the most likely mechanism by which *R. salmoninarum* was introduced is via live fish or egg movements from other countries. Phylogenetic reconstruction of a collection of *R. salmoninarum* from Chile, North America and Europe indicated that introductions have taken place repeatedly over the last 35 years, most likely via transfer of *Oncorhynchus* species and Atlantic salmon for stocking of rivers and aquaculture purposes. These data would suggest that the source of the introductions was most likely of North American and European origin, but that sample coverage in these regions was insufficient to confidently establish the exact sources of these introductions into Chile. Furthermore, two of the Chilean lineages are not closely related to any of the other clade 1 isolates, which suggests that the source of lineages 1c and 1d remained unsampled within the current collection. Historical records suggest that further sampling of both North American and Japanese isolates may help to identify the populations from which these endemic lineages arose.

The Chilean lineages have spread pervasively throughout the aquaculture production network in this country, with representative samples isolated from farms and hatcheries owned by multiple companies. We suggest that the observed phylogeny is not adequately explained by any single stage in the food production chain. This is likely to be a product of the spread of strains via the trade in eggs and contributed to by sub-lineages endemic to individual farms and hatcheries. These possibilities have important implications for understanding the spread of *R. salmoninarum* and the management of BKD in fish farming.

The Chilean lineages were found in multiple host species, but lineage 1c was significantly associated with a single host species, coho salmon. This lineage was predominantly sampled from sites owned by a single company, but may represent the early stages of adaptation to a host species, something not previously observed in *R. salmoninarum*.

This study suggests that *R. salmoninarum* has been introduced into Chile multiple times since the development of large-scale aquaculture. The presence of distinct, low-prevalence lineages, such as lineage 1d, may indicate that these introductions are common, but that these lineages fail to become established over large geographical ranges. The parity between the estimated introduction dates and key developments in the salmon industry would suggest that perturbations in the industry were formative events in the dissemination of these successful lineages throughout Chile. Potentially unsampled reservoirs of *R. salmoninarum* in the form of salmon that run the rivers in Chile should not be discounted as factors that have contributed to the longevity of these successful lineages. Further sampling of *R. salmoninarum* over a range of geographical locations, both within Chile and worldwide, and from wild host species is necessary to build a deeper understanding of the current and historical movements of *R. salmoninarum*.

## Data bibliography

1. Brynildsrud O, Feil EJ, Bohlin J, Castillo-Ramirez S, Colquhoun D *et al.* European Bioinformatics Institute (EBI) Short Read Archive. Accession numbers ERR327904 to ERR327971 inclusive (2014).

## Supplementary Data

Supplementary File 1

## References

[1] FAO (2016). The State of World Fisheries and Aquaculture 2016..

[2] Alvial A, Kibenge F, Forster J, Burgos JM, Ibarra R (2012). The Recovery of the Chilean Salmon Industry: the ISA Crisis and its Consequences and Lessons.

[3] Sanders JE, Barros MJ (1986). Evidence by the fluorescent antibody test for the occurrence of *Renibacterium salmoninarum* among salmonid fish in Chile. J Wildl Dis.

[4] SERNAPESCA. (2015.). Informe Sobre Uso de Antimicrobianos en la Salmonicultura Nacional 2015.

[5] Barnes AC, Delamare-Deboutteville J, Gudkovs N, Brosnahan C, Morrison R (2016). Whole genome analysis of *Yersinia ruckeri* isolated over 27 years in Australia and New Zealand reveals geographical endemism over multiple lineages and recent evolution under host selection. Microb Genom.

[6] Bayliss SC, Verner-Jeffreys DW, Bartie KL, Aanensen DM, Sheppard SK (2017). The promise of whole genome pathogen sequencing for the molecular epidemiology of emerging aquaculture pathogens. Front Microbiol.

[7] Brynildsrud O, Feil EJ, Bohlin J, Castillo-Ramirez S, Colquhoun D (2014). Microevolution of *Renibacterium salmoninarum*: evidence for intercontinental dissemination associated with fish movements. ISME J.

[8] Bethke J, Quezada J, Poblete-Morales M, Irgang R, Yáñez A (2017). Biochemical, serological, and genetic characterisation of *Renibacterium salmoninarum* isolates recovered from salmonids in Chile. Bull Eur Assoc Fish Pathol.

[9] SUBPESCA (2013). Resolución Exenta No. 1741. Establece Clasificación de Enfermedades de Alto Riesgo.

[10] SUBPESCA (2001). Reglamento de Medidas de Protección, Control y Erradicación de Enfermedades de Alto Riesgo para las Especies Hidrobiológicas.

[11] SERNAPESCA (2013). Aprueba Programa Sanitario General de Manejo de la Reproducción de Peces (PSGR).

[12] Evelyn TPT (1977). An improved growth medium for the kidney disease bacterium and some notes on using the medium. Bull Off Int Epizoot.

[13] Ponstingl H, Ning Z (2010). SMALT-a new mapper for DNA sequencing reads. F1000 Posters.

[14] McKenna A, Hanna M, Banks E, Sivachenko A, Cibulskis K (2010). The Genome Analysis Toolkit: a MapReduce framework for analyzing next-generation DNA sequencing data. Genome Res.

[15] Li H, Handsaker B, Wysoker A, Fennell T, Ruan J (2009). The Sequence Alignment/Map format and SAMtools. Bioinformatics.

[16] Connor TR, Loman NJ, Thompson S, Smith A, Southgate J (2016). CLIMB (the Cloud Infrastructure for Microbial Bioinformatics): an online resource for the medical microbiology community. Microb Genom.

[17] Kurtz S, Phillippy A, Delcher AL, Smoot M, Shumway M (2004). Versatile and open software for comparing large genomes. Genome Biol.

[18] Stamatakis A (2014). RAxML version 8: a tool for phylogenetic analysis and post-analysis of large phylogenies. Bioinformatics.

[19] Jombart T, Kendall M, Almagro-Garcia J, Colijn C (2017). treespace: statistical exploration of landscapes of phylogenetic trees. Mol Ecol Resour.

[20] Page AJ, Taylor B, Delaney AJ, Soares J, Seemann T (2016). SNP-sites: rapid efficient extraction of SNPs from multi-FASTA alignments. Microb Genom.

[21] Croucher NJ, Page AJ, Connor TR, Delaney AJ, Keane JA (2015). Rapid phylogenetic analysis of large samples of recombinant bacterial whole genome sequences using Gubbins. Nucleic Acids Res.

[22] Didelot X, Wilson DJ (2015). ClonalFrameML: efficient inference of recombination in whole bacterial genomes. PLoS Comput Biol.

[23] Yu G, Smith DK, Zhu H, Guan Y, Lam TT-Y (2017). ggtree: an r package for visualization and annotation of phylogenetic trees with their covariates and other associated data. Methods Ecol Evol.

[24] Wickham H (2009). Ggplot2: Elegant Graphics for Data Analysis.

[25] Rambaut A, Lam TT, Max Carvalho L, Pybus OG (2016). Exploring the temporal structure of heterochronous sequences using TempEst (formerly Path-O-Gen). Virus Evol.

[26] Bouckaert R, Heled J, Kühnert D, Vaughan T, Wu CH (2014). BEAST 2: a software platform for Bayesian evolutionary analysis. PLoS Comput Biol.

[27] Drummond AJ, Ho SY, Phillips MJ, Rambaut A (2006). Relaxed phylogenetics and dating with confidence. PLoS Biol.

[28] Argimón S, Abudahab K, Goater RJ, Fedosejev A, Bhai J (2016). Microreact: visualizing and sharing data for genomic epidemiology and phylogeography. Microb Genom.

[29] Duchêne S, Holt KE, Weill FX, Le Hello S, Hawkey J (2016). Genome-scale rates of evolutionary change in bacteria. Microb Genom.

[30] van Belkum A, Scherer S, van Alphen L, Verbrugh H (1998). Short-sequence DNA repeats in prokaryotic genomes. Microbiol Mol Biol Rev.

[31] United Nations (2006). A Case Study of the Salmon Industry in Chile.

[32] SUBPESCA (2018). Informe Sectorial Diciembre 2017.

[33] Godoy MG, Aedo A, Kibenge MJ, Groman DB, Yason CV (2008). First detection, isolation and molecular characterization of infectious salmon anaemia virus associated with clinical disease in farmed Atlantic salmon (*Salmo salar*) in Chile. BMC Vet Res.

[34] Vike S, Nylund S, Nylund A (2009). ISA virus in Chile: evidence of vertical transmission. Arch Virol.

