# Supplementary material for: Genomic epidemiology of the commercially important pathogen Renibacterium salmoninarum within the Chilean salmon industry
Source: Microb Genom. 2018 Jul 24;4(9):e000201. doi: 10.1099/mgen.0.000201 (PMC6202448; doi:10.1099/mgen.0.000201)
Supplement: Supplementary File 1 [file mgen-4-201-s001.pdf]

Supplementary Figure 1

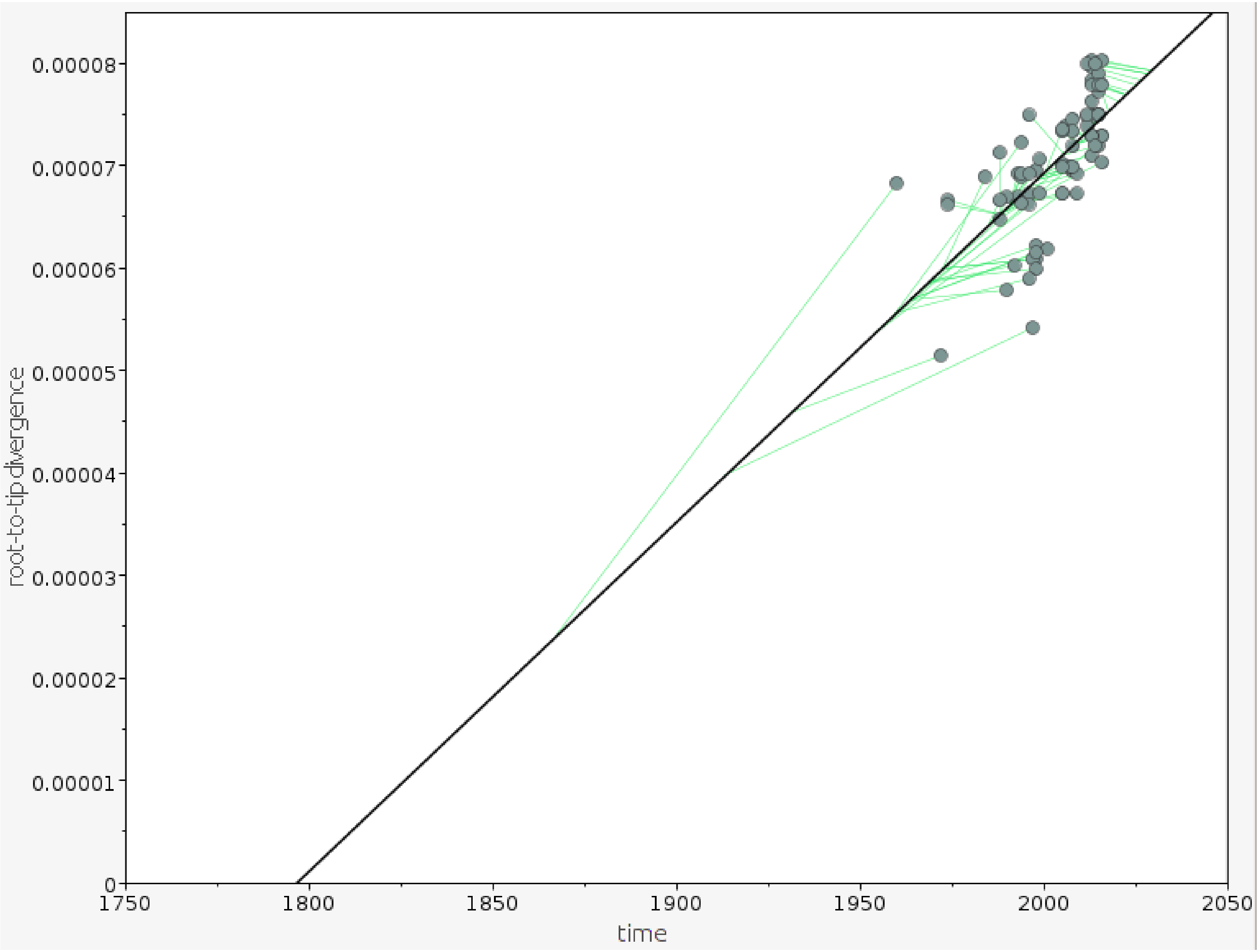

Supplementary Figure 2

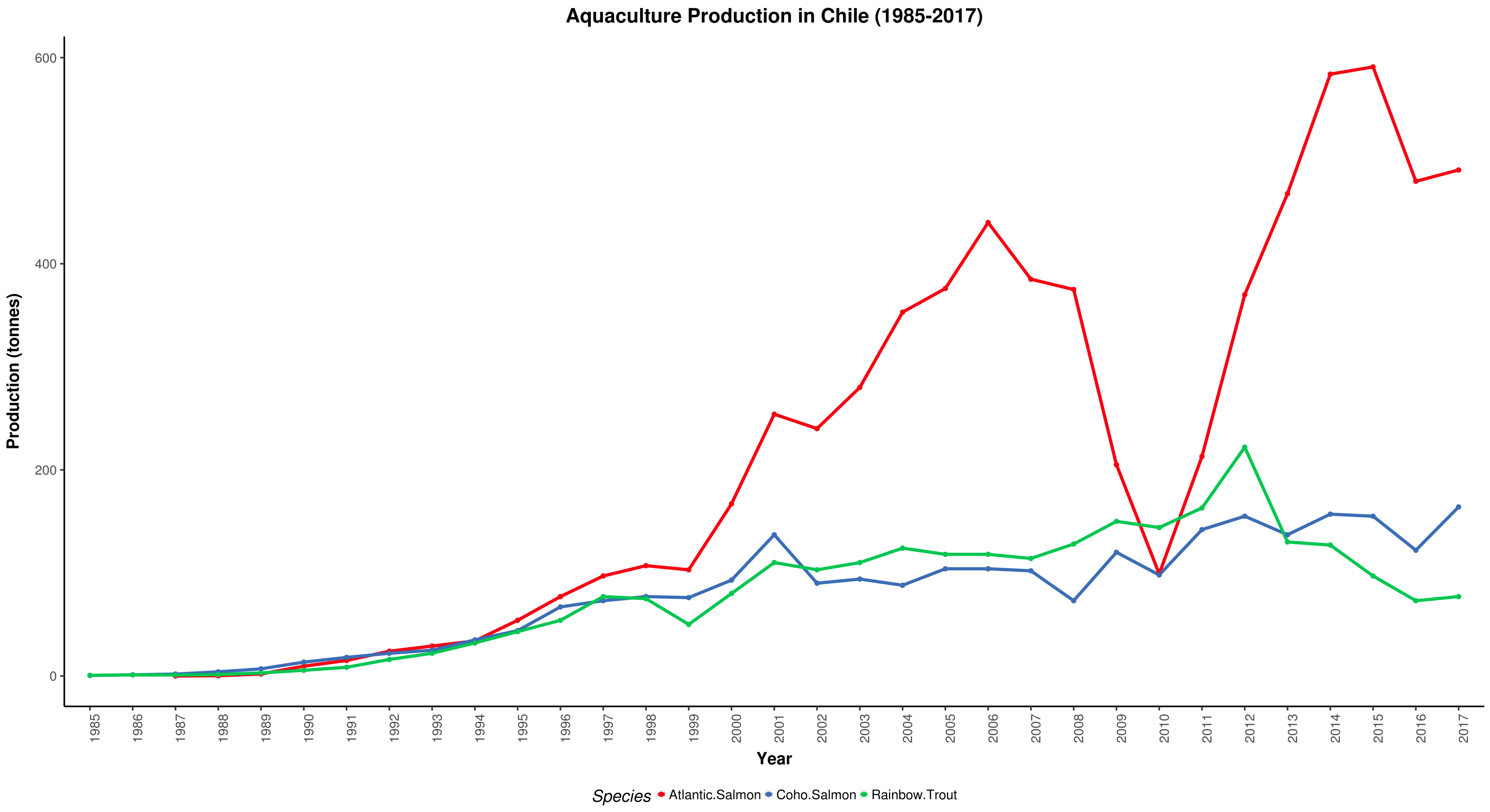

Supplementary Figure 3

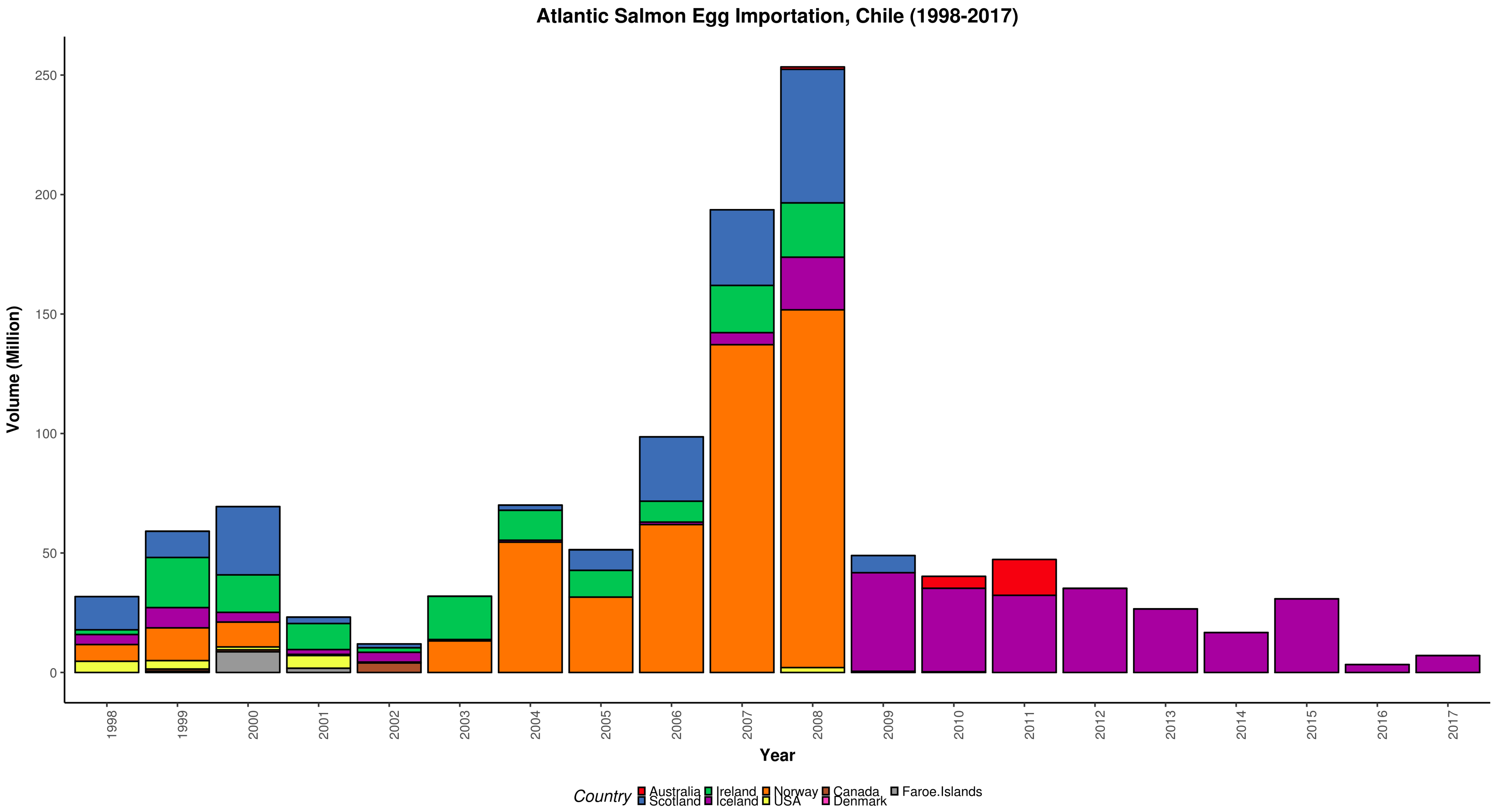

Supplementary Table 1

Fishers Exact **P-value before correction**

	Phenotype	*Lineage 1A*	*Lineage 1B*	*Lineage 1C*
Host Species	*S. salar*	0.000	0.163	0.007
	O. kisutch	1.000	1.000	0.000
	O. mykiss	0.002	0.447	0.446
Life Stage	*Broodstock*	0.608	0.561	1.000
	Fingerling	1.000	1.000	1.000
	Fry	0.489	1.000	1.000
	Smolt	1.000	0.660	0.404
	Adult	0.222	0.716	0.162
Region	*VIII*	0.476	1.000	1.000
	IX	0.135	1.000	0.086
	X	0.062	0.269	0.152
	XI	0.243	0.662	0.655
	XII	1.000	0.561	0.236
Salt/Fresh	*Salt water*	1.000	1.000	0.019
	Freshwater	1.000	1.000	0.019
Company	*Company 1*	1.000	1.000	1.000
	Company 2	1.000	1.000	1.000
	Company 3	1.000	1.000	1.000
	Company 4	0.001	1.000	0.001
	Company 5	0.007	0.232	0.134
	Company 6	1.000	1.000	1.000
	Company 7	0.099	0.007	1.000
	Company 8	0.489	1.000	1.000
	Company 9	1.000	1.000	1.000
	Company 10	0.476	0.214	1.000
Freshwater Site	*FW 1*	0.003	1.000	0.004
	FW 2	1.000	1.000	1.000
	FW 3	0.022	0.303	0.001
	FW 4	1.000	1.000	1.000
	FW 5	0.438	1.000	0.313
	FW 6	0.142	0.008	1.000
	FW 7	1.000	1.000	1.000
	FW 8	0.438	1.000	0.313
	FW 9	1.000	1.000	1.000
Point Of Sampling	*Farm site 1*	1.000	1.000	1.000
	Farm site 2	1.000	1.000	1.000
	Farm site 3	1.000	1.000	1.000
	Farm site 4	1.000	0.387	1.000
	Farm site 5	0.489	1.000	1.000
	Farm site 6	0.221	1.000	0.052
	Farm site 7	0.476	1.000	0.238
	Farm site 8	0.476	1.000	0.238
	Farm site 9	0.476	1.000	0.238
	Farm site 10	0.476	1.000	0.238
	Farm site 11	0.476	0.214	1.000
	Farm site 12	0.476	1.000	0.238
	Farm site 13	1.000	1.000	1.000
	Farm site 14	1.000	1.000	1.000
	Farm site 15	0.476	1.000	0.238
	Farm site 16	1.000	1.000	1.000
	Farm site 17	1.000	1.000	1.000
	Farm site 18	0.476	0.214	1.000
	Farm site 19	0.476	1.000	1.000
	Farm site 20	1.000	1.000	1.000
	Farm site 21	0.476	0.214	1.000
	Farm site 22	0.476	0.214	1.000
	Farm site 23	0.476	0.214	1.000
	Farm site 24	1.000	1.000	1.000
	Farm site 25	0.476	0.214	1.000
	FW 1	0.489	1.000	1.000
	FW 2	1.000	1.000	1.000
	FW 3	0.221	1.000	0.052
	FW 4	1.000	1.000	1.000
	FW 6	0.221	0.042	1.000
	FW 10	0.233	1.000	1.000
	FW 11	0.489	1.000	1.000
	FW 12	1.000	1.000	1.000

P-value after bonferroni correction

	Phenotype	*Lineage 1A*	*Lineage 1B*	*Lineage 1C*
Host Species	*S. salar*	0.006	1.000	0.171
	O. kisutch	1.000	1.000	0.000
	O. mykiss	0.037	1.000	1.000
Life Stage	*Broodstock*	1.000	1.000	1.000
	Fingerling	1.000	1.000	1.000
	Fry	1.000	1.000	1.000
	Smolt	1.000	1.000	1.000
	Adult	1.000	1.000	1.000
Region	*VIII*	1.000	1.000	1.000
	IX	1.000	1.000	1.000
	X	0.934	1.000	1.000
	XI	1.000	1.000	1.000
	XII	1.000	1.000	1.000
Salt/Fresh	*Salt water*	1.000	1.000	0.116
	Freshwater	1.000	1.000	0.116
Company	*Company 1*	1.000	1.000	1.000
	Company 2	1.000	1.000	1.000
	Company 3	1.000	1.000	1.000
	Company 4	0.044	1.000	0.025
	Company 5	0.222	1.000	1.000
	Company 6	1.000	1.000	1.000
	Company 7	1.000	0.220	1.000
	Company 8	1.000	1.000	1.000
	Company 9	1.000	1.000	1.000
	Company 10	1.000	1.000	1.000
Freshwater Site	*FW 1*	0.075	1.000	0.119
	FW 2	1.000	1.000	1.000
	FW 3	0.606	1.000	0.028
	FW 4	1.000	1.000	1.000
	FW 5	1.000	1.000	1.000
	FW 6	1.000	0.206	1.000
	FW 7	1.000	1.000	1.000
	FW 8	1.000	1.000	1.000
	FW 9	1.000	1.000	1.000
Point Of Sampling	*Farm site 1*	1.000	1.000	1.000
	Farm site 2	1.000	1.000	1.000
	Farm site 3	1.000	1.000	1.000
	Farm site 4	1.000	1.000	1.000
	Farm site 5	1.000	1.000	1.000
	Farm site 6	1.000	1.000	1.000
	Farm site 7	1.000	1.000	1.000
	Farm site 8	1.000	1.000	1.000
	Farm site 9	1.000	1.000	1.000
	Farm site 10	1.000	1.000	1.000
	Farm site 11	1.000	1.000	1.000
	Farm site 12	1.000	1.000	1.000
	Farm site 13	1.000	1.000	1.000
	Farm site 14	1.000	1.000	1.000
	Farm site 15	1.000	1.000	1.000
	Farm site 16	1.000	1.000	1.000
	Farm site 17	1.000	1.000	1.000
	Farm site 18	1.000	1.000	1.000
	Farm site 19	1.000	1.000	1.000
	Farm site 20	1.000	1.000	1.000
	Farm site 21	1.000	1.000	1.000
	Farm site 22	1.000	1.000	1.000
	Farm site 23	1.000	1.000	1.000
	Farm site 24	1.000	1.000	1.000
	Farm site 25	1.000	1.000	1.000
	FW 1	1.000	1.000	1.000
	FW 2	1.000	1.000	1.000
	FW 3	1.000	1.000	1.000
	FW 4	1.000	1.000	1.000
	FW 6	1.000	1.000	1.000
	FW 10	1.000	1.000	1.000
	FW 11	1.000	1.000	1.000
	FW 12	1.000	1.000	1.000

Supplementary Table 2

Divergence Dates

		Time since most recent sample			*Date*			Date Integer
**Strict Clock**	**Node/Group**	*Mean (height)*	*Higher (height_95%_HPD)*	*Lower (height_95%_HPD)*	*Mean*	*Higher*	*Lower*	*Mean*
	Lineage 1a	21.65	15.88	28.17	1994.35	2000.12	1987.83	1994
	Lineage 1b	7.97	5.30	11.04	2008.03	2010.70	2004.96	2008
	Lineage 1c	31.83	21.29	43.47	1984.17	1994.71	1972.53	1984
	Lineage 1b vs MT2943	100.77	79.06	124.14	1915.23	1936.94	1891.86	1915
	Lineage 1	385.52	302.57	476.92	1630.48	1713.43	1539.08	1630
	Lineage 1 vs Lineage 2	4076.11	3190.77	5042.24	-2060.11	-1174.77	-3026.24	-2060

		Time since most recent sample			*Date*			Date Integer
**Relaxed Clock**	**Node/Group**	*Mean (height)*	*Higher (height_95%_HPD)*	*Lower (height_95%_HPD)*	*Mean*	*Higher*	*Lower*	*Mean*
	Lineage 1a	19.98	12.61	27.77	1996.02	2003.39	1988.23	1996
	Lineage 1b	7.71	4.94	11.05	2008.30	2011.06	2004.95	2008
	Lineage 1c	30.00	13.64	48.96	1986.00	2002.37	1967.05	1986
	Lineage 1b vs MT2943	91.32	52.50	133.39	1924.68	1963.50	1882.61	1925
	Lineage 1	322.96	187.87	474.78	1693.04	1828.13	1541.22	1693
	Lineage 1 vs Lineage 2	2754.25	1065.87	4687.65	-738.25	950.13	-2671.65	-738

Mutation Sites

	Variable Sites	Genome Size (bp)	N Sites	Real Site No.	Prop. Variable
**Current**	3504	3155250	173674	2981576	0.0011752174
**Brynildsrud**	3600	3155250	-	3155250	0.0011409556

Mutations per year

		mutations/variable sites/year	mutations/genome/year
**Strict**	**clock.rate**	1.55E-04	1.82E-07
**Relaxed**	**ucld.Mean**	1.78E-04	2.09E-07
**Brynildsrud**	-	3.32E-04	3.79E-07
